# Salvianolate injection for hypertensive nephropathy patients who were using valsartan: A systematic review and meta-analysis

**DOI:** 10.3389/fphar.2023.1119150

**Published:** 2023-01-30

**Authors:** Qiyao Xu, Yuehong Shen, Jianqiao Zhao, Jianping Shen

**Affiliations:** ^1^ Affiliated Hospital of Integrated Traditional Chinese and Western Medicine, Nanjing University of Chinese Medicine, Nanjing, China; ^2^ Graduate School, Nanjing University of Chinese Medicine, Nanjing, China; ^3^ School of Chinese Medicine, School of Integrated Chinese and Western Medicine, Nanjing University of Chinese Medicine, Nanjing, China

**Keywords:** salvianolate, hypertensive nephropathy, meta–analysis, valsartan, systematic review

## Abstract

**Background:** The treatment of hypertensive nephropathy has remained unchanged for many years. Salvianolate is the main active component extracted from *Salvia Miltiorrhiza*. The current studies seem to suggest that salvianolate has a certain therapeutic effect on hypertensive nephropathy.

**Objective:** The purpose of this meta-analysis is to evaluate the effect and safety of salvianolate on hypertensive nephropathy under the condition of standardized use of valsartan.

**Methods:** We conducted a systematic search (unlimited initial date to 22 October 2022) in PubMed, Web of Science, the Cochrane Library, Embase, China National Knowledge Infrastructure, Wanfang Data knowledge service platform, China Science and Technology Journal Database, China Biomedical Literature Service System. Searching for the study of salvianolate on hypertensive nephropathy. Two reviewers independently included the study that met the inclusion criteria, and extracted data, evaluated the quality of the study. We use RevMan5.4 and stata15 software for this meta-analysis. We use GRADEprofiler 3.2.2 software for evidence quality assessment.

**Results:** This meta-analysis included seven studies (525 patients). Compared with the use of valsartan combined with conventional treatment, salvianolate combined with valsartan and conventional treatment can further improve the efficacy (RR = 1.28, 95%CI:1.17 to 1.39), reduce blood pressure [systolic blood pressure (MD = 8.98, 95%CI:−12.38 to −5.59); diastolic blood pressure (MD = 5.74, 95%CI:−7.20 to −4.29)], serum creatinine (MD = −17.32, 95%CI:−20.55 to −14.10), blood urea nitrogen (MD = −1.89, 95%CI:−3.76 to −0.01), urine microalbumin (MD = −23.90, 95%CI:−26.54 to −21.26), and urinary protein to creatinine ratio (MD = −1.92, 95%CI:−2.15 to −1.69), cystatin C (MD = −1.04, 95%CI: −1.63 to −0.45) and increase calcitonin gene-related peptide (MD = 18.68, 95%CI:12.89 to 24.46) without increasing adverse reactions (RR = 2.20, 95%CI:0.52 to 9.40). But it has no additional effect on endothelin-1 and malondialdehyde. The quality of evidence ranged from moderate to very low.

**Conclusion:** This meta-analysis shows that the salvianolate can further improve renal function of hypertensive nephropathy patients based on valsartan was used. Therefore, salvianolate can be used as a clinical supplement for hypertensive nephropathy. However, the quality of the evidence is not high due to the uneven quality of the included studies and the insufficient sample size, we still need a lot of large sample size studies with more perfect design to confirm these results.

**Systematic Review Registration**: https://www.crd.york.ac.uk/prospero/display_record.php?ID=CRD42022373256, identifier CRD42022373256

## 1 Introduction

It is estimated that about 30% of the general population worldwide suffers from hypertension, and hypertensive nephropathy (HN) is considered one of the consequences of uncontrolled hypertension over a long period (Costantino et al., 2021). Blood pressure control remains suboptimal in the modern world, as 25% of hypertensive patients do not achieve ideal blood pressure targets, which results in numerous patients with HN (Whelton et al., 2018; Kao and Huang, 2021). Following diabetic nephropathy, HN is one of the most common causes of end-stage kidney disease and chronic kidney disease (CKD) (Udani et al., 2011). CKD and hypertension usually occur in concomitant circumstances, but it may be difficult to determine which disease developed first. (Seccia et al., 2017). It is well accepted that each component of the renal system can be affected by high blood pressure: the vessels, glomeruli, and tubulointerstitial tissues. The capillaries tuft damage that causes sclerosis and hyalinosis of kidney, as well as the renin-angiotensin system, have been studied for a long time (Costantino et al., 2021). Many studies have investigated the molecular mechanisms and other histological aspects of the pathophysiology of hypertensive nephropathy, including tubular cell damage that induces epithelial-interstitial transition and tubulointerstitial fibrosis (Bakin et al., 2002; Patel et al., 2005; Ruiz-Ortega et al., 2007). Additionally, proteinuria, dyslipidemia, and smoking are also high risk factors for HN (Jafar et al., 2003; Jo et al., 2020; Kuma and Kato, 2022). If kidney function is impaired, BP may be more difficult to control than in people without kidney disease (Wiederkehr et al., 2005). Moreover, HN patients have higher cardiovascular mortality and higher risk of cardiovascular disease, such as myocardial infarction and heart failure (Mann et al., 2001; Weiss et al., 2015; Khayyat-Kholghi et al., 2021). However, HN and end-stage kidney disease are predicted to continue growing in the coming decades, owing to aging, and increased survival rates from cardiovascular diseases (Williams et al., 2018). Current studies suggest that active control of BP and reduction of urinary protein (UP) are the main goals of the treatment of HN (Wiederkehr et al., 2005). Hoping to protect residual nephrons with antihypertensive drugs and delay the progression of renal damage (Ott and Schmieder, 2022). In terms of drug therapy, angiotensin-converting enzyme inhibitors/angiotensin II–receptor blockers (ARBs) were still the first-line medication to decrease the BP and UP (Wiederkehr et al., 2005). Beyond the current treatment, we are still interested in preserving renal function, which happens to be the function of many traditional Chinese medicines.

Salvia miltiorrhiza (DanShen) is a commonly used traditional Chinese herbal medicine. It has been first recorded in *Shennong Herbal Classic*, and was listed as the top grade (Pu et al., 2021). Salvia miltiorrhiza decoction pieces and many extracts have been proven to be effective for many diseases, especially cardiovascular and cerebrovascular diseases (Hao et al., 2021). Animal experimentations have confirmed that multiple extracts of DanShen have an anti-inflammatory effect *in vivo* and *in vitro*. This effect mainly by suppressing the release of tumor necrosis factor-α (TNF-α), interleukin-6 (IL-6), IL-1β, and the expression of cyclooxygenase-2 and inducible nitric oxide synthase (Gao et al., 2017; Yuan et al., 2019). In addition, DanShen extracts can effectively ameliorate the renal clearance of mice (Gao et al., 2018), dose-dependently decreased UP, blood urea nitrogen (BUN), serum creatinine (Scr), plasma cholesterol, and triglycerides in rats. This can be attributable to the suppression of nuclear factor-κB and p38 mitogen-activated protein kinase signaling pathways by DanShen extract (Zhang H F et al., 2018). It can be seen that the anti-inflammatory effect of DanShen is one of the mechanisms of improving renal function. Otherwise, DanShen extracts can induce podocyte autophagy by inhibiting phosphatidylinositol 3-kinase/protein kinase B/mammalian target of a rapamycin signaling pathway to make renal function better and reduce pathological injury in mice with nephrotic syndrome (Chen et al., 2022), can also reduce BP in rats through inhibiting angiotensin-converting enzyme and relaxing vascular smooth muscle (Kang et al., 2002; Zhang X D et al., 2018). In clinical research, many studies have confirmed that salvianolate can lower BP and improve renal function-related indicators (Fu et al., 2012), significantly reduce Scr, BUN and 24-hour UP in CKD patinets (Zhang et al., 2022).

As previously mentioned, valsartan, as an ARBs, is currently recognized as a first-line medication for the HN (Unger et al., 2020). Salvianolate, in combination with valsartan, has been shown to have a positive effect on HN in numerous studies. As a result of salvianolate treatment, BP can be further reduced, renal function is improved, the inflammatory response can be inhibited, etc. However, the sample sizes included in the current studies were small, and there was not enough evidence to confirm the clinical effect of salvianolate. Therefore, we conducted an assessment about whether salvianolate is an effective complementary therapy for HN under the premise of valsartan-included conventional treatment by a meta-analysis.

## 2 Methods

We have registered this system review and published the protocol on International Prospective Register of Systematic Reviews (PROSPERO) before we started retrieval studies, and we have completed this systematic review in accordance with this protocol. The registration number is CRD42022373256.

### 2.1 Literature search

We performed a systematic search (unlimited initial date to 22 October 2022) in the following databases: PubMed, the Cochrane Library, Embase, Web of Science, China National Knowledge Infrastructure (CNKI), Wanfang Data knowledge service platform (Wanfang Data), China Science and Technology Journal Database (VIP), and China Biomedical Literature Service System (SinoMed). The search strategy we used in the English database was Salvia* AND hypertens* AND (renal OR nephropathy OR kidney), and in the Chinese database was danshenduofen AND gaoxueya AND (shenbing OR shensunhai OR shenzangbing OR shenzangsunhai) (Supplementary Tables S1, S2). The retrieval scope was all fields included MeSH and Emtree. There was a restriction on the retrieval language to Chinese and English.

### 2.2 Study selection

Two authors (QX and YS) independently included or excluded the retrieved literature according to the following criteria.

Included studies must met each of the following inclusion criteria: 1) The patients included in the study met the accepted diagnostic criteria for HN, such as those specified in clinical guidelines, World Health Organization criteria, authoritative works, and clinical medical textbooks; 2) The study was a published clinical randomized controlled study; 3) The intervention factors of treatment group included salvianolate and valsartan with combined with conventional treatment which refers to other treatment for NH and treatment for patients’ original diseases in accordance with medical principles; 4) The intervention factors of control group included valsartan combined with conventional treatment; 5) The duration of treatment did not exceed 2 weeks according to the drug instructions of salvianolate injection; 6) The outcomes of the study reported included one or more of BP, renal function (Scr, BUN, urine microalbumin, Cystatin C, Urine protein to creatinine ratio), inflammatory factors (TNF-α, hypersensitive C-reactive protein and IL-6), oxidative stress indicators (malondialdehyde, glutathione peroxidase, and superoxide dismutase), factors affecting vasodilatory state (endothelin-1 and calcitonin gene-related peptide), clinical efficacy, and adverse reactions. BP, renal function and clinical efficacy were the primary outcomes. The other outcomes were the secondary outcomes. Clinical efficacy refers to whether the patient’s condition is improved judged by researcher according to the clinical performance. It is effective if the condition is improved, otherwise it is invalid.

Studies that meet any of the following criteria will be excluded: 1) The design of the study was seriously flawed; 2) Sufficient valid data could not be secured; 3) All data was published repeatedly.

### 2.3 Quality assessment

Two authors (QX and JZ) independently assessed the degree of bias risk of methodological of included studies with the RavMan5.4 software from the Cochrane Collaboration. A consultation was conducted when there was a disagreement between the two assessments. If there was still a disagreement, the third author (JS) will make the final decision.

### 2.4 Data extraction

Two authors (QX and YS) independently extracted data from each study according to excel spreadsheet designed in advance based on our research proposal. The data extracted included name of authors, publication year, condition of grouping, basic information about the sample (size, age, gender, etc.,), course of disease, intervention methods, and outcome indicators. A third reviewer (JS) was included for arbitration purposes. We contacted the corresponding author for confirmation when the data in the study was unclear, not detailed, or some studies were duplicated.

### 2.5 Statistical analysis

The risk ratio was selected as the effect quantity of the dichotomous variables, the standard mean difference or mean difference as the effect quantity of the continuous variables (Quinn et al., 2021). The synthesis will be displayed by forest plot. The interval estimation was expressed using 95% confidence intervals (95%CI), and set the significance level as *p* <0.05 (Xu M et al., 2017). We used the chi-square test based on Cochran’s Q test and I^2^ statistic to assess the heterogeneity of the studies. Based on the severity of the heterogeneity, I^2^≥50% is generally considered indicative of large heterogeneity, a random effect model was selected, and heterogeneity analysis is required (Xu Q et al., 2017). Otherwise, a fixed effect model was sued for data synthesis (Xu M et al., 2017). The publication bias was analyzed and evaluated by egger’s test (Xu Q et al., 2017). The sensitivity analysis was conducted by the one-by-one elimination method and was used to evaluate the robustness of the meta-analysis results (Crocerossa et al., 2021). When there were multiple CKD stages (According to Kidney Disease Outcomes Quality Initiative/DOQI) in one data synthesis, and at least one CKD stage contains two or more studies, a subgroup analysis based on CKD stage would be performed. The forest plot and heterogeneity analysis were performed by using RevMan (Version 5.4.1, The Cochrane Collaboration, 2020), egger’s test and sensitivity analysis were analyzed by using Stata 15.0 (Stata Corp, College Station, TX, United States).

### 2.6 Evidence quality assessment

Two authors (QX and YS) independently evaluated the quality of each result from five aspects of risk of bias, inconsistency, indirectness, imprecision, and publication bias by GRADEprofiler 3.2.2 software according to GRADE Handbook (Guyatt et al., 2008). Four different levels of evidence quality may be obtained through assessment: high, moderate, low and, very low (Guyatt et al., 2008). If there are differences in the evaluation process, they should be resolved through negotiation. If the negotiation cannot be resolved, the third author should make the final decision.

## 3 Results

### 3.1 Search results and study characteristics

**FIGURE 1:**
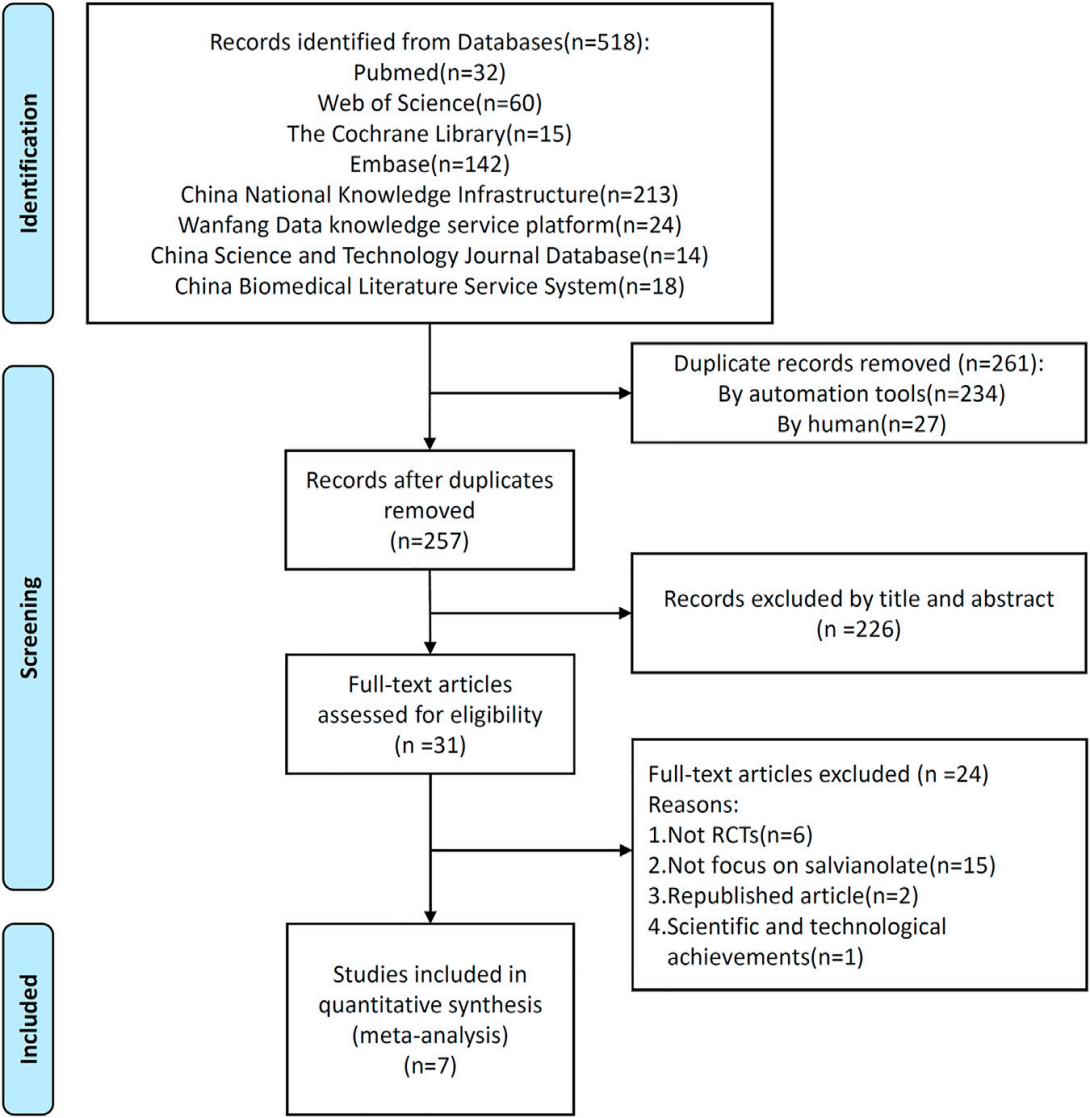
Flow diagram of literature screening.

We retrieved 518 studies from both Chinese and English databases, and finally included seven randomized controlled trials (Wang et al., 2015; Wang J, 2015; Wang et al., 2016; Ding, 2017; Wang et al., 2017; Liu, 2018; Wang, 2018) for systematic review and meta-analysis (Figure 1). These studies, published between 2015 and 2018, were all from China and included a total of 535 patients, of whom 266 were in the treatment group. Two studies (Wang et al., 2015; Wang et al., 2016) may be different parts of the same study, and we calculated the sample size and the outcome only once. The same outcome indicators were subject to newly published articles of these two studies. We tried to contact the author of both two studies *via* email, but there was no response. The characteristics of included studies were summarized in Table 1.

**TABLE 1:** Characteristics of included studies.

Study	Sample size,sex (M/F), age (year, mean ± SD)		Duration of HBP (year, mean ± SD)	Diagnostic criteria of HN	eGFR (mL/min)	Intervention		Course of treatment	Outcome	Adverse reactions
					or CKD staging	T	C			
wang,2018	T:45 (25/20,56.48 ± 7.39)		T:13.92 ± 5.92	1999 WHO-ISH diagnostic criteria (Chalmers, 1999)	—	Salvianolate (200 mg, ivgtt, qd)+ valsartan (80 mg, po, qd)	valsartan (80 mg, po, qd)	14 days	Curative effect, Scr, BUN, Malb, ET-1, IL-6, TNF-α, MDA	Not reported
	C:48 (26/22,55.27 ± 7.93)		C:13.69 ± 5.68							
liu,2018	T:53 (31/22,61.9 ± 7.40)		T:12.2 ± 2.5	1999 WHO-ISH diagnostic criteria (Chalmers, 1999)	—	Salvianolate (200 mg, ivgtt, qd)+ valsartan (80 mg, po, qd)	valsartan (80 mg, po, qd)	14 days	Curative effect, Cys-C, Scr, UP/UCr	Not reported
	C:53 (30/23,61.4 ± 6.6)		C:12.4 ± 2.6							
ding,2017	T:40 (25/15,60.08 ± 5.19)<		T:9.54 ± 3.05	*Nephrology* (Wang et al., 1996)	T:35.02 ± 16.57	Salvianolate (200 mg, ivgtt, qd)+ valsartan (80 mg, po, qd)	valsartan (80 mg,po,qd)	14 days	Curative effect, BP, Cys-C, Scr, UP/UCr	Not reported
	C:40 (24/16,61.15 ± 5.23)		C:10.14 ± 3.53		C:34.91 ± 17.14					
wang, zheng et al., 2017	T:38	(42/34,56.8 ± 7.2)	—	*Nephrology* (Wang et al., 1996)	C:CKD I-II	Salvianolate (200 mg, ivgtt, qd)+ valsartan (80 mg, po, qd)	valsartan (80 mg, po, qd)	14 days	Curative effect, BP, Cys-C, Scr, UP/UCr	T:4 cases
	C:38				T:CKD I-II					C:2 cases
wang, wei et al., 2016	T:45 (25/20,56.8 ± 3.2)		T:9.8 ± 3.1	*Nephrology* (Wang et al., 1996)	T:34.7 ± 16.9	Salvianolate (100 mg, ivgtt, qd)+ valsartan (80 mg, po, qd)	valsartan (80 mg, po, qd)	14 days	Curative effect, BP, Cys-C, Scr, UP/UCr	T:2
	C:45 (26/19,57.2 ± 3.0)		C:9.7 ± 3.2		C:34.9 ± 16.5					C:none
wang, pan et al., 2015	T:45 (25/20,56.8 ± 3.2)		T:10 ± 3	*Nephrology* (Wang et al., 1996)	T:35 ± 17	Salvianolate (100 mg, ivgtt, qd)+ valsartan (80 mg, po, qd)	valsartan (80 mg, po, qd)	14 days	Curative effect, BP, Cys-C, Scr, UP/UCr, ET-1, CGRP	Not reported
	C:45 (26/19,57.2 ± 3.0)		C:10 ± 3		C:35 ± 16					
wang,2015	T:45		—	*Nephrology* (Wang et al., 1996)	—	Salvianolate (100 mg, ivgtt, qd)+ valsartan (80 mg, po, qd)	valsartan (80 mg, po, qd)	14 days	Curative effect, BP, Cys-C, Scr, BUN, Urine β2-Mglb, Urine Malb, 24hUP, ET-1, CGRP, MDA	Not reported
	C:45									

T, treatment group; C, control group; M, male; F, female; HBP, hypertension; HN, hypertensive nephropathy; WHO-ISH, World Health Organization and the International Society of Hypertension; Scr, serum creatinine; SD, standard deviation; BP, blood pressure; BUN, blood urea nitrogen; Malb, microalbumin in urine; UP/UCr, Urine protein/Urine creatinine; ET-1, endothelin-1; IL-6, interleukin-6; TNF-α, tumor necrosis factor-α; MDA, malondialdehyde; Cys-C, Cystatin C; SBP, systolic blood pressure; DBP, diastolic blood pressure; CKD, chronic kidney disease; CGRP, calcitonin gene-related peptide; Mglb, microglobulin; 24hUP, 24-hour urinary protein.

### 3.2 Risk of bias in included studies

**FIGURE 2:**
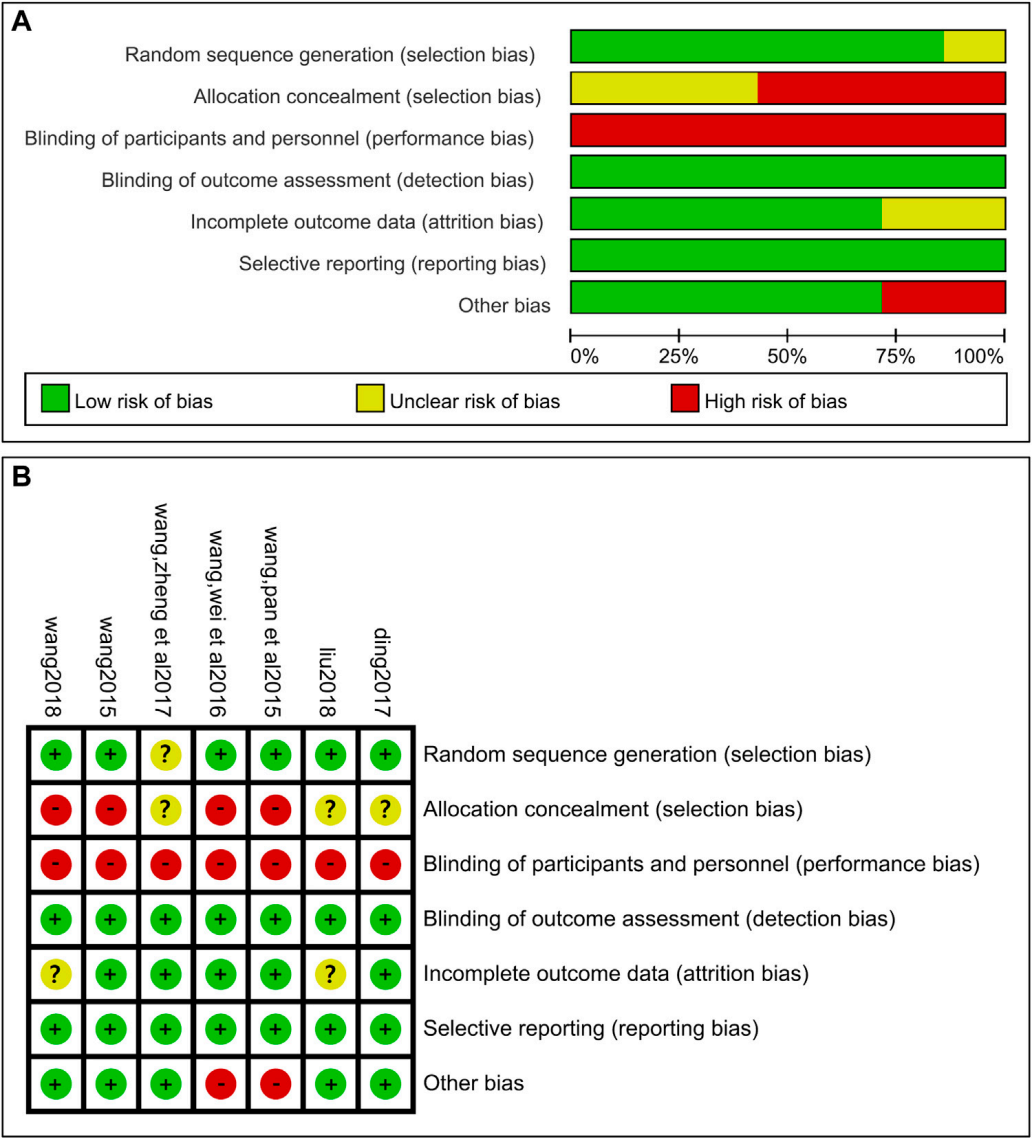
Risk of bias graph **(A)**. Risk of bias summary **(B)**.

Among all the studies, six studies (Wang et al., 2015; Wang J, 2015; Wang et al., 2016; Ding, 2017; Liu, 2018; Wang, 2018) explicitly reported randomization, but the randomization method did not explicitly indicate hiding. None of the studies used blinding for researchers and patients. All studies did not adopt blinding of outcome assessment, but the system reviewer judged that the outcome measurement did not be affected by the unblinded method. Therefore, the project of blinding the outcome evaluators is low risk. All studies reported complete outcome data without selective reporting. Part of the data of two studies (Wang et al., 2015; Wang et al., 2016) were identical, which belongs to repeated publication bias. The specific quality assessment was shown in Figure 2.

### 3.3 Outcome measures

#### 3.3.1 Clinical efficacy

**FIGURE 3:**
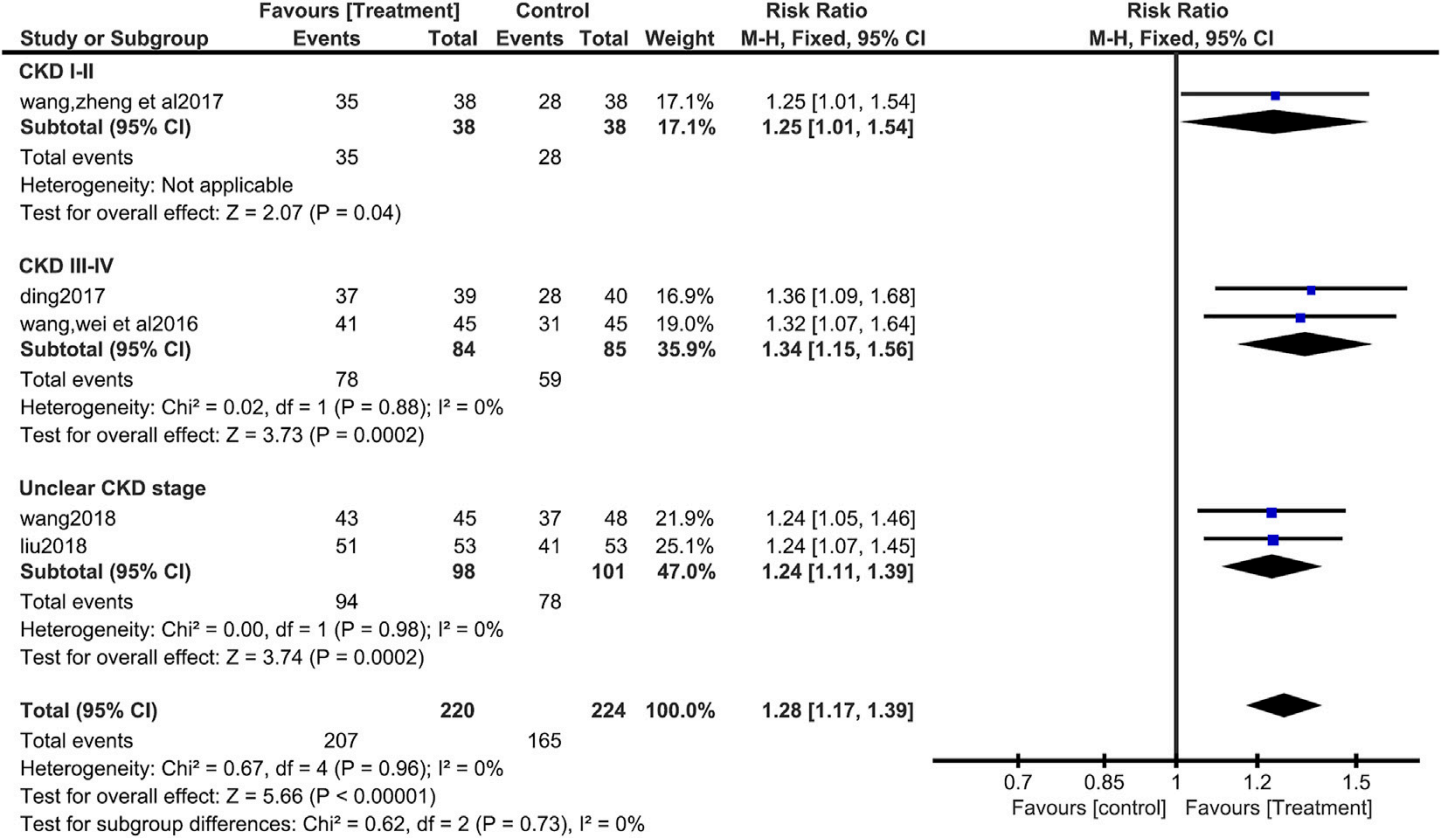
Forest plot for subgroup analysis of clinical efficacy between the treatment group and control group.

Five studies (Wang et al., 2016; Ding, 2017; Wang et al., 2017; Liu, 2018; Wang, 2018) (445 patients) evaluated clinical efficiency. There was no heterogeneity in the studies (I^2^ = 0.00%, *p* = 0.955), fixed effect model analysis showed that compared with valsartan-included conventional treatment group, the clinical efficacy of the group of salvianolate with valsartan-included conventional treatment was better (RR = 1.28, 95%CI:1.17 to 1.39, Z = 5.66, *p* <0.00001) (Figure 3). The subgroup analysis was performed based on the stage of CKD, and the included studies were divided into group stage I-II, group stage III-IV, and group of unclear stage. Subgroup analysis found no difference between the subgroups (Test for subgroup differences: I^2^ = 0%, *p* = 0.73) (Figure 3).

#### 3.3.2 Blood pressure

**FIGURE 4:**
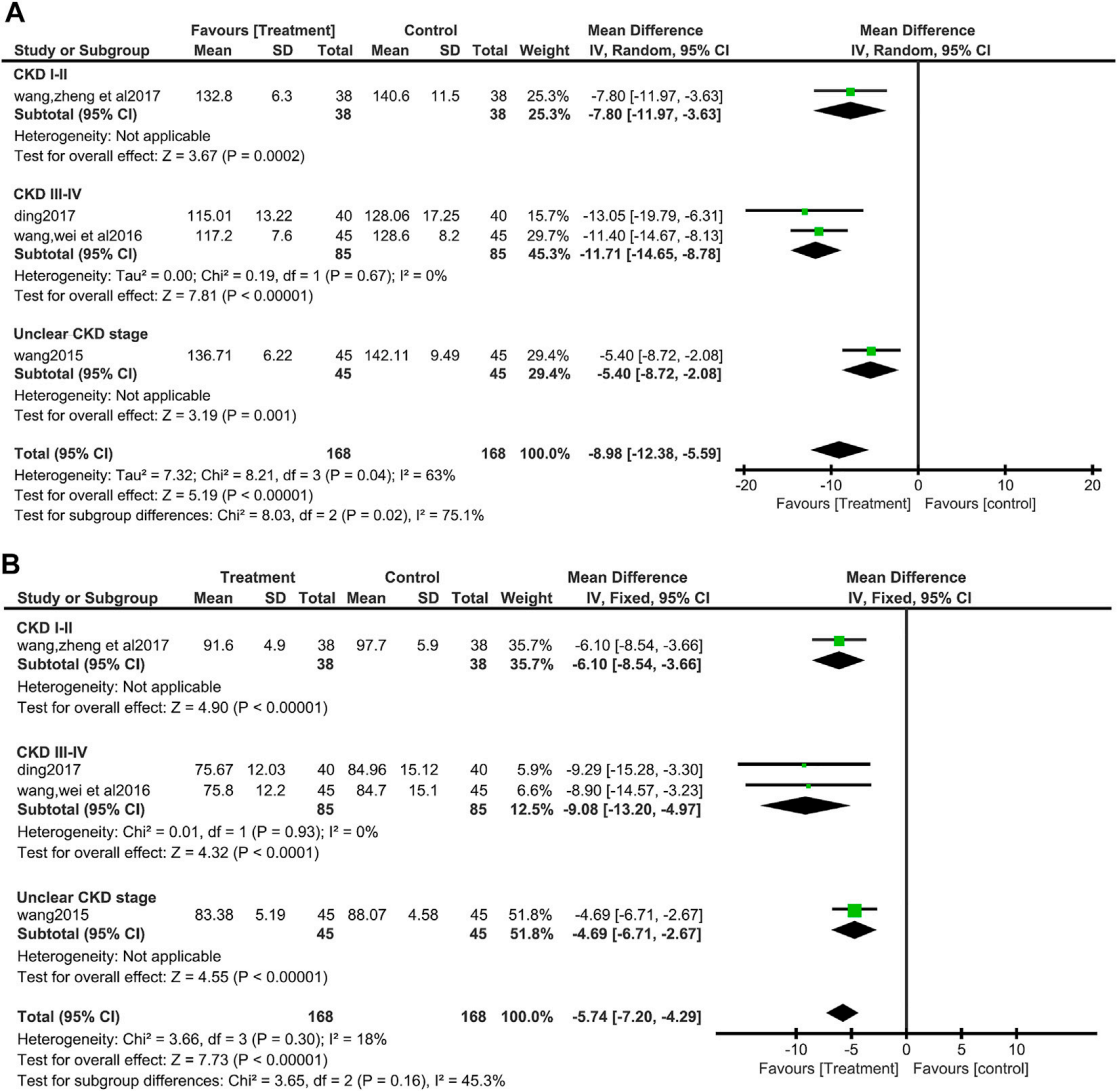
Forest plot for subgroup analyse of systolic blood pressure between the treatment group and control group **(A)**. Forest plot for subgroup analyse of diastolic blood pressure between the treatment group and control group **(B)**.

There were four studies (Wang J, 2015; Wang et al., 2016; Ding, 2017; Wang et al., 2017) (336 patients) that measured BP, both systolic and diastolic. For systolic BP(SBP), Due to the significant heterogeneity (I^2^ = 63%, *p* = 0.04), the random effects model was chosen, and the forest plots showed that compared with control group, SBP of the treatment group decreased more significantly (MD = 8.98, 95%CI:−12.38 to −5.59, Z = 5.19, *p* <0.00001) (Figure 4A). No significant heterogeneity was found in the data synthesis of diastolic blood pressure (I^2^ = 18%, *p* = 0.30). Fixed effect model analysis showed that salvianolate combined with the valsartan-included western medicine group had a more obvious DBP reduction (MD = 5.74, 95%CI:−7.20 to −4.29, Z = 7.73, *p* <0.00001) (Figure 4B). No publication bias was found by egger’s test for diastolic blood pressure (t = −0.57, *p* = 0.623) (Supplementary Figure S1). In subgroup analyses based on the stage of CKD, there were differences in SBP reduction between subgroups (Test for subgroup differences: I^2^ = 75.1%, *p* = 0.02), heterogeneity within subgroups (CKD III-IV) disappeared (I^2^ = 0%, *p* = 0.67), and DBP reduction between subgroups did not differ (Test for subgroup differences: I^2^ = 45.3%, *p* = 0.16) (Figures 4A, B).

#### 3.3.3 Renal function

##### 3.3.3.1 Serum creatinine

**FIGURE 5:**
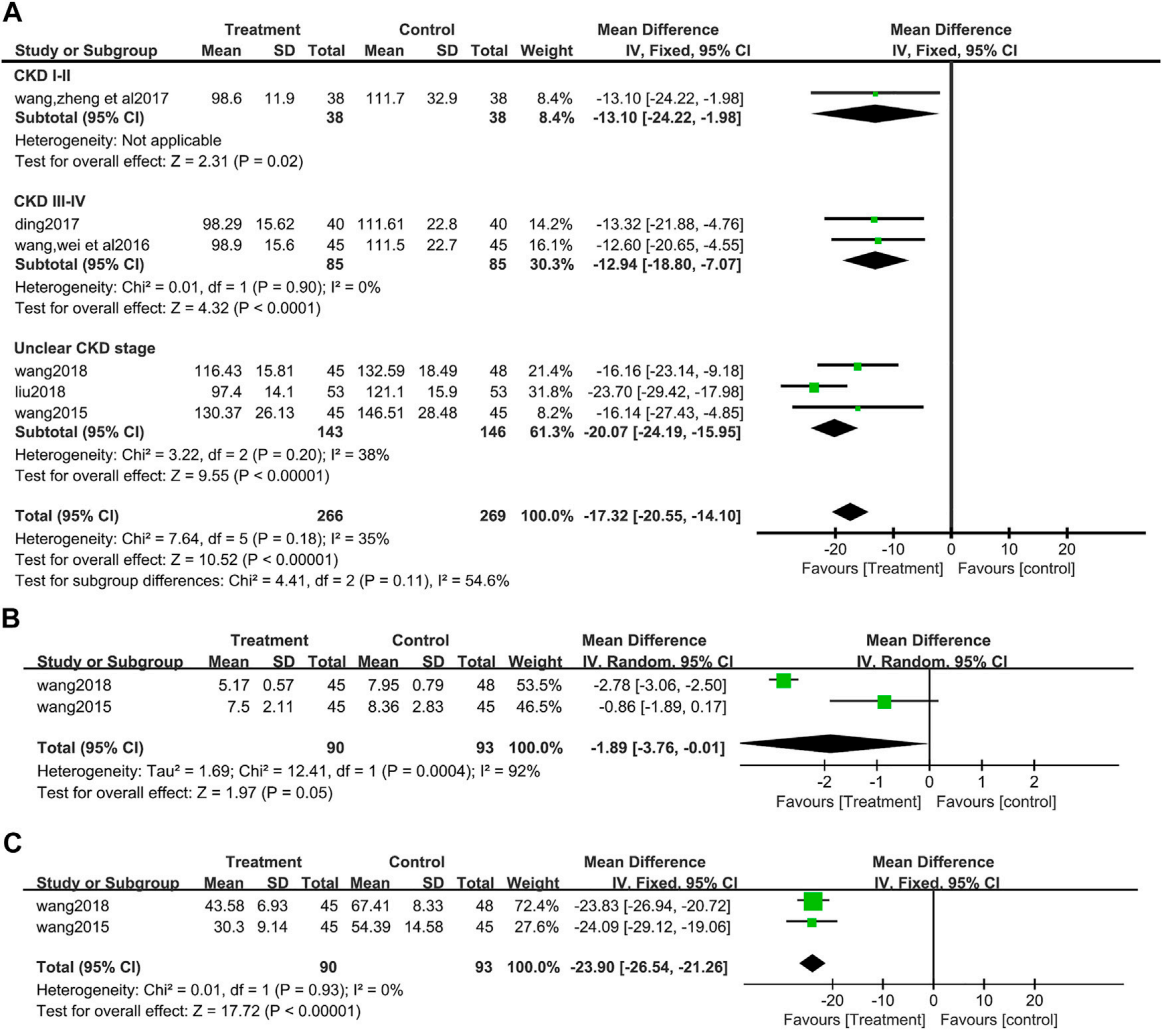
Forest plot for subgroup analyse of serum creatinine between the treatment group and control group **(A)**. **(B)** Forest plot for blood urea nitrogen between the treatment group and control group **(B)**. **(C)** Forest plot for urine microalbumin between the treatment group and control group **(C)**.

A total of six studies (Wang J, 2015; Wang et al., 2016; Ding, 2017; Wang et al., 2017; Liu, 2018; Wang, 2018) (495 patients) reported Scr values. There was no obvious heterogeneity among the six studies, according to heterogeneity analysis (I^2^ = 35%, *p* = 0.18). Fixed effects model analysis showed that salvianolate combined with the valsartan group had a better effect on reducing Scr (MD = −17.32, 95%CI:−20.55 to −14.10, Z = 10.52, *p* <0.00001) (Figure 5A). Differences among the subgroup were found by subgroup analysis based on CKD stage (Test for subgroup differences: I^2^ = 54.6%, *p* = 0.11), but differences mainly occurred in the unclear CKD stage group, and the heterogeneity increased in this group, while the heterogeneity of CKD III-IV group disappeared (I^2^ = 0%, *p* = 0.90) (Figure 5A).

##### 3.3.3.2 Blood urea nitrogen

After an analysis of two studies (Wang J, 2015; Wang, 2018) (183 patients) that reported BUN, significant heterogeneity was found between studies (I^2^ = 92%, *p* = 0.0004), and a random effect model analysis showed that compared with the valsartan group, salvianolate combined with valsartan could better reduce BUN (MD = −1.89, 95%CI:−3.76 to −0.01, Z = 1.97, *p* = 0.05) (Figure 5B).

##### 3.3.3.3 Urine microalbumin (UMAlb)

There were two studies (Wang J, 2015; Wang, 2018) (183 patients) that reported UMAlb. There was no heterogeneity in these two studies (I^2^ = 0%, *p* = 0.93), then a data synthesis by a fixed effect model showed that the decrease of UMAlb was more obvious in the salvia miltiorrhiza polyphenol acid group (MD = −23.90, 95%CI:−26.54 to −21.26, Z = 17.72, *p* <0.00001) (Figure 5C).

##### 3.3.3.4 Urine protein to creatinine ratio (UPCR)

**FIGURE 6:**
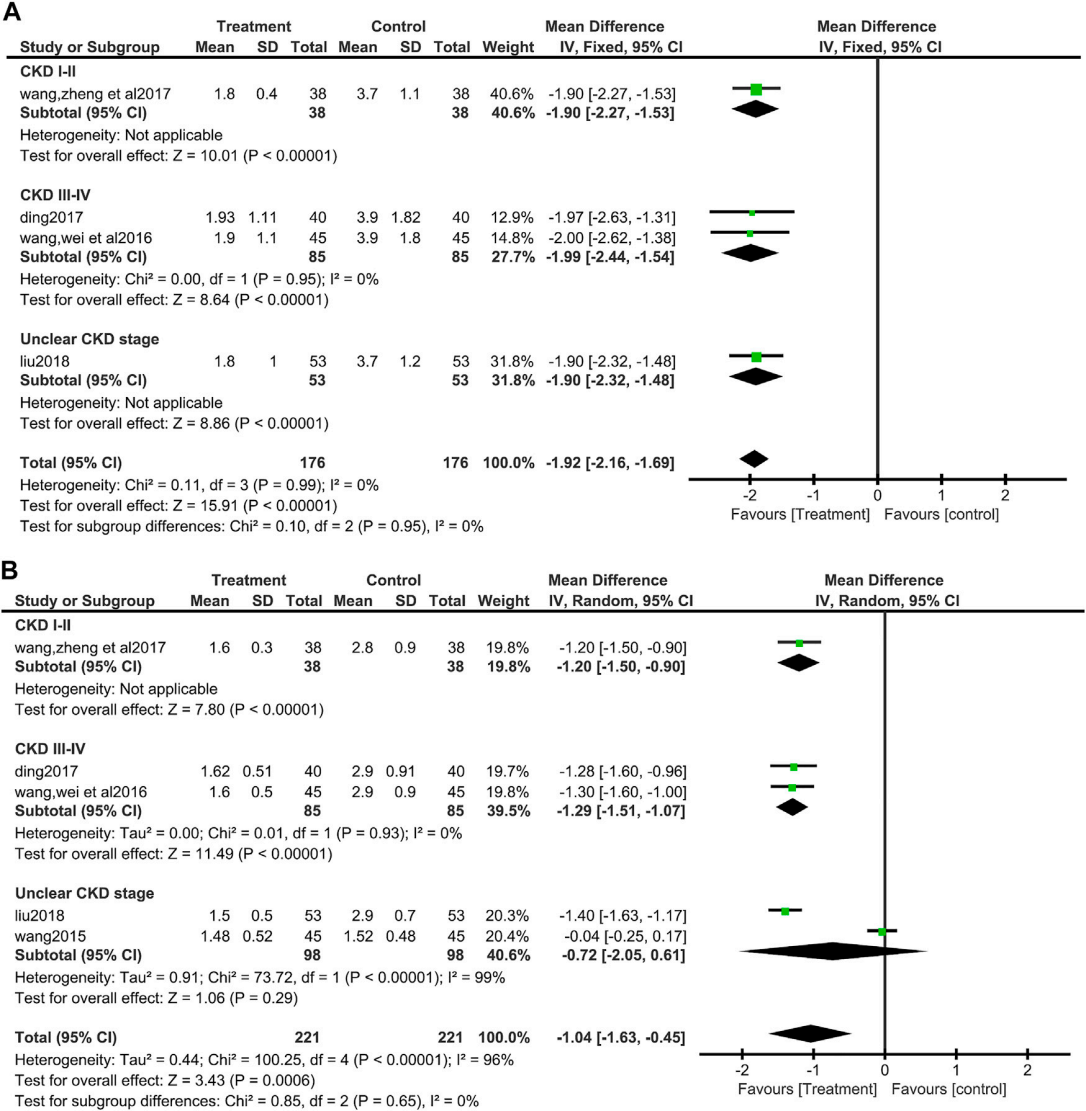
Forest plot for urine protein to creatinine ratio between the treatment group and control group **(A)**. Forest plot for subgroup analyse of Cystatin C between the treatment group and control group **(B)**.

Four of the included studies (Wang et al., 2016; Ding, 2017; Wang et al., 2017; Liu, 2018) (352 patients) showed the value of UPCR. No heterogeneity was found between these four included studies (I^2^ = 0%, *p* = 0.99). Data synthesis and subgroup analysis by fixed effect models showed that patients who used salvianolate had a more significant reduction in UPCR than those who did not use salvianolate (MD = −1.92, 95%CI:−2.15 to −1.69, Z = 15.91, *p* <0.00001), and there was no difference among subgroups based on the stage of CKD (I^2^ = 0%, *p* = 0.95) (Figure 6A).

##### 3.3.3.5 Cystatin C (Cys-C)

A total of five studies (Wang J, 2015; Wang et al., 2016; Ding, 2017; Wang et al., 2017; Liu, 2018) (442 patients) reported Cys-C values. Due to the significant heterogeneity among the studies involved in data synthesis (I^2^ = 96%, *p* <0.00001), so a random effect model was selected for processing the data. The analysis showed that salvianolate combined with the valsartan group had a better effect on reducing Cys-C (MD = −1.04, 95%CI:−1.63 to −0.45, Z = 3.43, *p* <0.0006) (Figure 6B). Egger’s test found no publication bias (t = −0.57, *p* = 0.623) (Supplementary Figure S2). The subgroup analysis based on the CKD stage found no difference among the subgroups (Test for subgroup differences: I^2^ = 0%, *p* = 0.65), but the heterogeneity of CKD III-IV group disappeared (I^2^ = 0%, *p* = 0.93), while the heterogeneity increased in unclear CKD stage group (I^2^ = 99%, *p* = 0.00001), and the MD value of this subgroup becomes no longer statistically significant (MD = −0.72, 95%CI:−2.05 to 0.61, Z = 1.06, *p* = 0.29) (Figure 6B).

#### 3.3.4 Vasodilation regulated factors

In the seven included studies, endothelin-1 and calcitonin gene-related peptide has been reported two or more times, and data can be synthesized.

##### 3.3.4.1 Endothelin-1 (ET-1)

**FIGURE 7:**
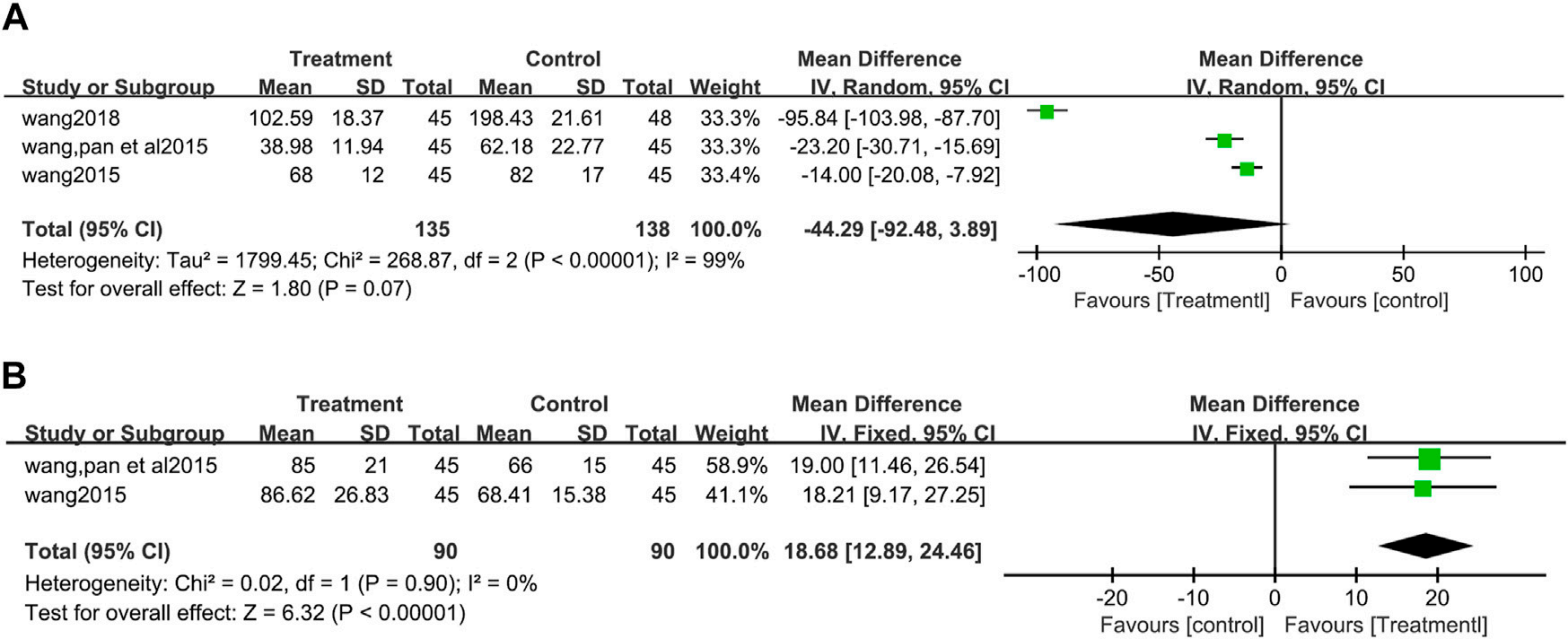
Forest plot for Endothelin-1 between the treatment group and control group Figure **(A)**. Forest plot for calcitonin gene-related peptide between the treatment group and control group **(B)**.

There was significant heterogeneity among three studies (Wang et al., 2015; Wang J, 2015; Wang, 2018) (273 patients) which reported ET-1 (I^2^ = 99%, *p* <0.00001). The egger’s test suggested that there was no publication bias (t = −1.35, *p* = 0.407) (Supplementary Figure S3). Random effects model was used for data synthesis, and the results showed no significant difference in the influence of the two groups on ET-1 (MD = −44.29, 95%CI: −92.48 to 3.89, Z = 1.80, *p* = 0.07) (Figure 7A).

##### 3.3.4.2 Calcitonin gene-related peptide (CGRP)

CGRP values were reported in two studies (Wang et al., 2015; Wang J, 2015) and no heterogeneity between the two studies (I^2^ = 0%, *p* = 0.90). The fixed effect model analysis showed that CGRP increased more significantly in patients used salvianolate (MD = 18.68, 95%CI:12.89 to 24.46, Z = 6.32, *p* <0.00001) (Figure 7B).

#### 3.3.5 Oxidative stress indicators

**FIGURE 8:**
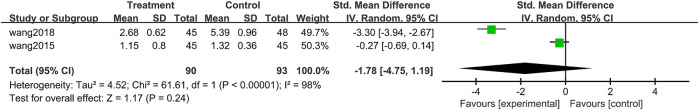
Forest plot for malondialdehyde between the treatment group and control group.

Among the included studies, three studies reported oxidative stress indicators, including glutathione peroxidase, malondialdehyde (MDA), nicotinamide adenine dinucleotide phosphate oxidase, and reactive oxygen species, among which MDA was reported in two studies (Wang et al., 2015; Wang J, 2015) and other indicators were reported only once. Data synthesis was conducted for the studies that reported MDA. The random effect model analysis showed that the difference between the two groups in the impact on MDA was no statistically significant (MD = −1.78, 95%CI: −4.75 to 1.19, Z = 1.17, *p* = 0.24) (Figure 8).

#### 3.3.6 Inflammatory factor

One study (Wang, 2018) out of seven included studies reported the effects on inflammatory factors, which did not meet the requirements for data synthesis. This study (Wang, 2018) has shown that salvianolate can significantly reduce the levels of inflammatory factors (IL-6 and TNF-α) in HN patients.

#### 3.3.7 Adverse reactions

**FIGURE 9:**
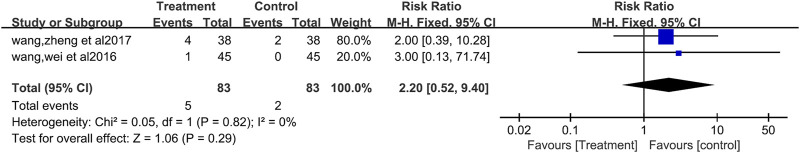
Forest plot for adverse reactions between the treatment group and control group.

Only 2 of 7 the included studies (Wang et al., 2016; Wang et al., 2017) reported adverse reactions that occurred during the study, including dizziness and headache, which were transient discomfort and did not affect the treatment. No heterogeneity was found in the data synthesis of adverse reactions (I^2^ = 0%, *p* = 0.82). Fixed effects model analysis showed that the difference between the two groups on adverse reactions was no statistical significance (RR = 2.20, 95%CI:0.52 to 9.40, Z = 1.06, *p* = 0.29) (Figure 9).

### 3.4 Publication bias

We conducted egger’s test for the studies that reported SBP, Cys-C, and ET-1 to analyze publication bias. Meta-analysis of these indicators showed significant heterogeneity among studies, and each meta-analysis included more than two studies. No publication bias was found in all three egger’s tests (Supplementary Figures S1–S3).

### 3.5 Sensitivity analysis

Sensitivity analysis was conducted by eliminating the included methods one by one to observe whether there were significant changes in the results to evaluate whether the results of our meta-analysis were robust (Table 2; Supplementary Figures S4–S10). As shown in the figures and table, the results of meta-analyses are robust. Excluding any studies, the overall trend of data synthesis has not changed, except for the ET-1 results of Wang’s study (Wang, 2018). Except for the Cys-C results of Wang’s study (Wang L, 2015) and the SBP result of Wang’s study (Wang L, 2015), the heterogeneity of other results did not change with the elimination of any study.

**TABLE 2:** Sensitivity analyses for Clinical Efficacy,SBP,DBP,Scr, UPCR,Cys-C,ET-1.

Indicators	Study	effect quantity with study omitted RR/MD/SMD (95%CI)	I^2^	P
Clinical Efficacy	none	1.28 (1.17, 1.39)	0%	0.96
	Liu,2018	1.29 (1.17, 1.43)	0%	0.91
	Wang,2018	1.29 (1.17, 1.42)	0%	0.91
	Ding,2017	1.26 (1.15, 1.38)	0%	0.97
	Wang et al. (2017)	1.28 (1.17, 1.41)	0%	0.89
	Wang et al. (2016)	1.27 (1.16, 1.39)	0%	0.92
SBP	none	-8.98 (-12.38, -5.59)	63%	0.04
	Ding (2017)	-8.23 (-11.92, -4.53)	69%	0.04
	Wang et al. (2017)	-9.53 (-14.30, -4.76)	75%	0.02
	Wang et al. (2016)	-7.86 (-11.58, -4.13)	51%	0.13
	Wang L (2015)	-10.40 (-13.15, -7.65)	18%	0.29
DBP	none	-5.74 (-7.20, -4.29)	18%	0.3
	Ding (2017)	-5.52 (-7.02, -4.02)	10%	0.33
	Wang et al. (2017)	-5.54 (-7.36, -3.73)	43%	0.17
	Wang et al. (2016)	-5.52 (-7.03, -4.01)	16%	0.3
	Wang L (2015)	-6.87 (-8.97, -4.78)	0%	0.47
Scr	none	-17.32 (-20.55, -14.10)	35%	0.18
	Liu (2018)	-14.35 (-18.26, -10.44)	0%	0.96
	Wang (2018)	-17.64 (-21.28, -14.00)	47%	0.11
	Ding (2017)	-17.99 (-21.47, -14.50)	40%	0.16
	Wang et al. (2017)	-17.71 (-21.08, -14.34)	43%	0.13
	Wang et al. (2016)	-18.23 (-21.75, -14.71)	34%	0.19
	Wang L (2015)	-18.45 (-22.16, -14.75)	49%	0.12
UPCR	none	-1.92 (-2.16, -1.69)	0%	0.99
	Liu (2018)	-1.93 (-2.22, -1.65)	0%	0.96
	Wang et al. (2017)	-1.94 (-2.25, -1.63)	0%	0.96
	Ding (2017)	-1.92 (-2.17, -1.66)	0%	0.96
	Wang et al. (2016)	-1.91 (-2.17, -1.65)	0%	0.98
Cys-C	none	-1.04 (-1.63, -0.45)	96%	< 0.00001
	Liu (2018)	-0.95 (-1.65, -0.25)	96%	< 0.00001
	Wang et al. (2017)	-1.00 (-1.73, -0.27)	97%	< 0.00001
	Ding (2017)	-0.98 (-1.70, -0.27)	97%	< 0.00001
	Wang et al. (2016)	-0.98 (-1.69, -0.26)	97%	< 0.00001
	Wang L (2015)	-1.31 (-1.45, -1.17)	0%	0.77
ET-1	none	-44.29 (-92.48, 3.89)	99%	< 0.00001
	Wang (2018)	-18.32 (-27.32, -9.32)	71%	0.06
	Wang et al. (2015)	-54.87 (-135.08, 25.33)	100%	< 0.00001
	Wang L (2015)	-59.50 (-130.69, 11.68)	99%	< 0.00001

RR, risk ratio; MD, mean difference; SMD, standard mean difference; SBP, systolic blood pressure; DBP, diastolic blood pressure; Scr, serum creatinine; UPCR, urine protein to creatinine ratio; Cys-C, cystatin C; ET-1, endothelin-1.

### 3.6 Evidence quality

We used the GRADE evidence quality rating to evaluate the evidence quality of the results of this meta-analysis. Among them, the quality of evidence of the effect of salvianolate on Scr in patients with hypertensive nephropathy is moderate; The clinical efficacy, adverse reactions, DBP, UMAlb, Cys-C, UPCR, and CGRP were low quality evidence; The quality of evidence on the impact of SBP, BUN, ET-1, and MDA was very low (Table 3).

**TABLE 3:** Evidence quality of the outcomes of HN patients treated with salvianolate.

Outcomes	No of studies	Risk of bias	Inconsistency	Indirectness	Imprecision	Publication bias	Quality
CE	5	serious^a^ ^,^ ^b^	no	no	serious^d^	none	low
SBP	4	serious^a^ ^,^ ^b^	serious^c^	no	serious^d^	none	very low
DBP	4	serious^a^ ^,^ ^b^	no	no	serious^d^	none	low
Scr	6	serious^a^ ^,^ ^b^	no	no	no	none	moderate
UMAlb	2	serious^a^ ^,^ ^b^	no	no	serious^d^	none	low
Cys-C	5	serious^a^ ^,^ ^b^	serious^c^	no	no	none	low
BUN	2	serious^a^ ^,^ ^b^	serious^c^	no	serious^d^	none	very low
UPCR	4	serious^a^ ^,^ ^b^	no	no	serious^d^	none	low
ET-1	3	serious^a^ ^,^ ^b^	serious^c^	no	very serious^d^ ^,^ ^e^	none	very low
CGRP	2	serious^a^ ^,^ ^b^	no	no	serious^d^	none	low
MDA	2	serious^a^ ^,^ ^b^	serious^c^	no	very serious^d^ ^,^ ^e^	none	very low
AR	2	serious^a^ ^,^ ^b^	no	no	serious^d^	none	low

HN, hypertensive nephropathy; CE, clinical efficacy; SBP, systolic blood pressure; DBP, diastolic blood pressure; Scr, serum creatinine; BUN, blood urea nitrogen; UMAlb, urine microalbumin; UPCR, urinary proteinuria creatinine ratio; Cys-C, Cystatin C; ET-1, Endothelin-1; CGRP, calcitonin gene-related peptide; MDA, malondialdehyde; AR, adverse reactions.

^a^ Inadequate allocation concealment.

^b^ Missing of blinding of participants and personnel.

^c^ Significant heterogeneity between studies.

^d^ The total sample size of the studies is less than 400.

^e^ The result is not significant.

## 4 Discussion

This systematic review included a total of 7 randomized controlled studies with 535 participants and is the first to specifically and comprehensively analyze the effect of salvianolate on the treatment of HN on the basis of standardized use of ARB drugs. This meta-analysis suggests that compared with valsartan alone combined with conventional western medicine, the addition of salvianolate can further improve the overall clinical efficacy, more effective in reducing BP, decrease Scr, BUN, UMAlb, UPCR, Cys-C, further increase of CGRP, no additional effect on ET-1, and MDA, no additional adverse reactions.

Controlling BP by various means is still an effective strategy to delay the progression of HN (Member of Chinese expert consensus group on diagnosis and treatment of hypertensive nephropathy, 2022). Lowering BP significantly delay the decline of GFR in CKD patients (Lee et al., 2021). The results of our meta-analysis are similar to those of previous studies, suggested that salvianolate can effectively reduce BP. Yang and Tang’s studies (Yang et al., 2012; Tang et al., 2020) have shown that salvianolate and other salvia miltiorrhiza extract can assist in the treatment of hypertension and further reduce systolic and DBP on the basis of the original treatment. Meng’s study (Meng et al., 2014) confirmed that salvianolate can inhibit arteriosclerosis in rats, which is the basis of HN (Udani et al., 2011; Dong et al., 2021). Salvianolate can inhibit microvascular remodeling and intimal hyperplasia caused by hypertension (Teng, 2015; Zhao, 2018), thereby reducing microvascular remodeling, target organ damage, and slow down the progress of HN. Although there was heterogeneity in the synthesis of SBP, we considered that the heterogeneity is related to the differences in CKD stages of patients included in various studies, the disappearance of heterogeneity after subgroup analysis confirms this conjecture, and the sensitivity analysis indicates that the results are robust. Based on the current results, we believe that salvianolate can be used to assist in controlling the BP of HN patients.

During the diagnosis and treatment of HN, various indicators of renal function are the top priority of the monitored items. Glomerular filtration rate (GFR), Scr, BUN, UP, Cys-C, etc., are all such indicators. Albuminuria is an early manifestation of CKD, and the presence of elevated creatinine or decreased glomerular filtration rate indicates that nephron loss has occurred (Levey et al., 2022). So controll UP is one of the main goals of HN treatment (Member of Chinese expert consensus group on diagnosis and treatment of hypertensive nephropathy, 2022). From our meta-analysis, we can see that the addition of salvianolate on the basis of valsartan can further reduce Scr, BUN, UMAlb, UPCR, and Cys-C. A systematic review analysis also points out that salvianolate can reduce Scr, BUN, UP, Cys-C, etc., of CKD, without obvious adverse reactions (Zhang et al., 2022). Therefore, salvianolate can improve the renal function of HN patients. However, in subgroup analysis of Cys-C, the result of unclear CKD stages subgroup suggested that salvianolate can not further reduce Cys-C. This is different from the overall result. We could not find convincing reasons for this, and the huge heterogeneity between the two studies does not have a confirmed source. We speculate that this may be related to the difference in age, CKD staging and other aspects between the patients included in the two studies, because neither of the two studies described the CKD stage, Wang’s article did not describe the patient’s age. But this conjecture cannot be confirmed at present. Therefore, the evidence quality of this result was reduced, and further research is needed to confirm it. The specific mechanism of salvianolate improving renal function still needs further study. Some studies have confirmed that salvianolate can reduce the apoptosis of renal podocytes (Liang et al., 2021). Others have found that salvianolate can inhibit the apoptosis of ischemic kidney cells by activating the kelch-like erythroid cell-derived protein with cap ‘N’ collar homology-associated protein 1-nuclear factor erythroid 2-related factor 2-antioxidant response element (Keap1-Nrf2-ARE) signal pathway (Sun et al., 2022). These may be the mechanism of salvianolate to improve renal function, but more in-depth research is still needed.

Oxidative stress and inflammatory reaction are the key factors in the process of renal damage caused by hypertension, and they can promote each other (Crowley, 2014). Therefore, antioxidant stress and anti-inflammatory reaction may be the key to delay the progress of HN. Previous studies have also confirmed that salvianolate can increase plasma CGRP (Hong et al., 2002; Versmissen et al., 2019), which agrees with meta-analysis. In addition, many studies have confirmed that salvia miltiorrhiza polyphenolic acid salt can reduce oxidative stress reaction. For example, salvia miltiorrhiza polyphenolate can increase the activity of superoxide dismutase and thioredoxin, reduce the content of MDA and active oxygen, can inhibit inducible nitric oxide synthase, and increase the activity of thioredoxin (Han et al., 2011; Fei et al., 2013; Zhang, 2017; Tang et al., 2020). The above effects may be related to the down-regulation of Smad2/3 and transforming growth factors beta 1 expression, increase of B-cell lymphoma 2/ B-cell lymphoma 2-Associated X ratio, and activation of Keap1-Nrf2-ARE signal pathway by salvianolate (Udani et al., 2011; Fei et al., 2013; Qiu et al., 2018; Sun et al., 2022). The change of MDA in our meta-analysis result is not significantly reduced as shown in previous studies, but has a downward trend and no statistical significance. However, as there are only two studies included, the heterogeneity between the studies is high, and no source of heterogeneity was found finally, so it is necessary to be cautious to take this as evidence. The role of salvianolic acid salt on oxidative stress still needs more research to enrich the evidence.

Hypertension can lead to the increase of vasoconstrictors (such as ET-1) and the decrease of vasodilators (such as nitric oxide), further leading to the increase of renal vascular resistance and promoting renal vascular remodeling, which is also one of the mechanisms of hypertension leading to kidney disease (Xu M et al., 2017; Versmissen et al., 2019). The effect of salvianolate on reducing ET-1 has been reported in many studies, and these studies show that salvianolate can inhibit vascular intima hyperplasia and remodeling (Xu et al., 2001; Wang L, 2015; Zhao, 2018; Zhu et al., 2020). But in our meta-analysis, it seems that after the use of valsartan, adding salvianolate has no additional effect on reducing ET-1. We can not deny that salvianolate has the effect of reducing ET-1, because valsartan has the effect of significantly reducing ET-1 (Mitchell et al., 2006; Li et al., 2010), so this effect of salvianolate may be masked by the effect of valsartan. The three studies included in the analysis had high heterogeneity, but no publication bias. The sensitivity analysis suggested that the heterogeneity came from Wang’s study (Wang, 2018), and the meta-analysis results changed significantly after excluding this study, suggesting that salvianolate could significantly reduce ET-1, so we considered that the meta-analysis results were not robust, and we need to treat this result with caution before there are more reliable data to confirm.

According to our meta-analysis, the effect of salvianolate on HN is definite, especially in improving renal function. Even if valsartan has been used, it can still further improve renal function without increasing adverse reactions. We graded the quality of evidence of our results using the GRADE approach. In the evaluation of evidence quality, only Scr is moderate, and others are in low and very low quality, which indicates that the overall quality of evidence of this meta-analysis is not high. The main reasons for the degradation of the quality of evidence are inadequate allocation concealment, missing of blinding of participants and personnel and insufficient sample size (Table 3). At the same time, we noted that the current studies in this field are all about short-term injection used in hospitalized patients without follow-up, so it is unclear how long the curative effect of salvianolate can last and whether it is only a short-term temporary effect. Therefore, we believe that based on this meta-analysis, there is sufficient evidence to support the clinical use of salvianolate to further improve renal function and further control BP in HN patients, especially in reducing Scr, and it can be safely used in patients with CKD1-4. Future research about salvianolate and HN should focus on high-quality long-term curative effect observation, to determine whether salvianolic acid salt can effectively delay the progress of kidney disease. High quality mainly requires attention to the use of allocation concealment, blinding and sufficient sample size. In addition, to clarify the relevant mechanism through basic research, so as to make further research on the vasodilation and on the inhibitory effect of oxidative stress of salvianolate in hypertensive nephropathy.

## 5 Limitations

Some limitations remain with this meta-analysis. First, although we developed a detailed search strategy, due to language limitations, we only searched in English and Chinese, and all included studies are in Chinese. As a result, we cannot guarantee an absence of language bias. Secondly, the overall quality of the included studies is not very high. All the studies did not accurately describe whether to use blinding, which undoubtedly increased the risk of performance bias. Some of the included studies do not clearly define the renal function stage of patients, so our subgroup analysis can only be conducted for the CKD III-IV stage. Fourth, the meta-analysis of CGRP and MDA showed significant heterogeneity, and the subgroup results of Cys-C were inconsistent. But we have not found convincing concrete reasons. It may be related to the sample size, course of the disease, CKD stage, etc., but the number of studies is insufficient, and the data provided by the study is limited, so we failed to conduct further analysis to find out the reason. Fifth, HN is a chronic disease, but all the studies we include do not carry out long-term follow-up and do not evaluate the long-term effect. Sixth, the results of this meta-analysis, as evidence, are generally of low quality. Finally, although several studies have been included, the overall sample size is still insufficient in this meta-analysis, and there are no relevant clinical studies after 2018 after the retrieval. Therefore, more new and large sample clinical studies are needed to provide data support.

## 6 Conclusion

As discussed in this meta-analysis, patients with HN can still benefit from salvianolate even if they have been treated regularly with valsartan. Salvianolate can still further improve efficacy, reduce BP and improve renal function, but the effectiveness of this treatment in further reducing oxidative stress and improving endothelial function needs to be confirmed. At the same time, we need to see that the quality of evidence of other results is very low except for Scr. In general, salvianolate can be used as an auxiliary treatment drug for HN, but further randomized controlled trials with large sample sizes, multi-centers, and double-blinding are needed to provide more reliable and accurate data to assess the efficacy of salvianolate for HN.

## Data Availability

The original contributions presented in the study are included in the article/Supplementary Material, further inquiries can be directed to the corresponding author.
