# Supplementary material for: Salvianolate injection for hypertensive nephropathy patients who were using valsartan: A systematic review and meta-analysis
Source: Front Pharmacol. 2023 Jan 30;14:1119150. doi: 10.3389/fphar.2023.1119150 (PMC9922779; doi:10.3389/fphar.2023.1119150)
Supplement: Supplementary file 1 [file DataSheet1.ZIP › Excluded studies after full text screening.docx]

Excluded studies after full text screening

| Reason of excluded | Study (original language) | Study (English) |
| --- | --- | --- |
| Not RCT | (刘翠华, 2013; 刘云豹, 2015; 王强等., 2016; 吐逊姑丽?木沙, 2017; 刘淑环 and 何宜汀, 2020; 陈凯丽等., 2022) | (Liu, 2013; Liu, 2015; Wang et al., 2016; Tu, 2017; Liu and He, 2020; Chen et al., 2022) |
| Not focus on salvianolate | (朱宏文等., 2011; 杨海燕等., 2012; 李玉洁, 2014; 张建女, 2014; 倪海涛, 2015; 李双喜等., 2016; 李婷, 2016; 王梦华, 2016; 楚召旭等., 2017; 呼思毓, 2017; 李强虎 and 郭杰, 2017; 马文涛等., 2017; 刘凤芝, 2018; 吕志广, 2018; 林燕, 2020) | Zhu et al., 2011; Yang et al., 2012; Li, 2014; Zhang, 2014; Ni, 2015; Li et al., 2016; Li, 2016; Wang, 2016; Chu et al., 2017; Hu, 2017; Li and Guo, 2017; Ma et al., 2017; Liu, 2018; Lv, 2018; Lin, 2020) |
| Republished | (王琳琳等., 2016; 李双喜, 2018) | (Wang et al., 2016; Li, 2018) |
| Scientific and technological achievements | (王琳琳等., 2015) | (Wang et al., 2015) |

**REFERENCES (original language)**

陈凯丽, 张单华, 徐培聪, 王报林, 邓伟. (2022). 中医药治疗高血压早期肾损害的用药规律. *中国医药导报* 19, 118-122.

楚召旭, 陈志君, 姜新杰, 胡夏敏. (2017). 丹参多酚盐联合前列地尔、谷胱甘肽对高血压早期肾损伤疗效观察. *心脑血管病防治* 17, 378-380. doi: 10.3969/j.issn.1009_816x.2017.05.17

呼思毓. (2017). 丹参酮联合缬沙坦对高血压肾病患者肾功能及炎症反应的影响. *中西医结合研究* 9, 239-240. doi: 10.3969/j.issn.1674-4616.2017.05.005

李强虎, 郭杰. (2017). 丹参酮注射液联合缬沙坦对高血压肾损害患者的影响. *新疆医学* 47, 1304-1305,1308.

李双喜. 丹参多酚酸盐、前列地尔、谷胱甘肽联合应用治疗慢性肾功能不全的疗效分析. 中国中西医结合学会肾脏疾病专业委员会2018年学术年会. 其他:海军军医大学附属长海医院,2018. 1366.

李双喜, 郭志勇, 李娟, 赵丽芳. (2016). 丹参多酚酸盐、前列地尔、谷胱甘肽联合应用治疗慢性肾衰竭的疗效分析. *中国中西医结合肾病杂志* 17, 789-790.

李婷. (2016). 丹参酮联合缬沙坦治疗高血压肾病的效果观察. *世界临床医学* 10, 88.

李玉洁. (2014). 丹参酮注射液联合缬沙坦治疗高血压肾损害115例临床观察. *中国民族民间医药* 57-57,58.

林燕. (2020). 丹参多酚酸盐、前列地尔、谷胱甘肽联合应用治疗慢性肾功能衰竭的临床效果观察. *中国医药指南* 18, 172-173.

刘翠华. (2013). 中西医结合对难治性高血压的肾脏损害防护以及护理. *时珍国医国药* 24, 2275-2276. doi: 10.3969/j.issn.1008-0805.2013.09.107

刘凤芝. (2018). 高血压肾病的临床特点及丹参酮注射液联合缬沙坦治疗效果评估. *中西医结合心血管病电子杂志* 6, 74. doi: 10.3969/j.issn.2095-6681.2018.13.052

刘淑环, 何宜汀. (2020). 丹参注射液联合西药对难治性高血压的肾脏损害的防护以及护理干预研究. *首都食品与医药* 27, 130. doi: 10.3969/j.issn.1005-8257.2020.02.105

刘云豹. (2015). 丹参多酚酸盐治疗慢性肾功能衰竭16例. *中国中医药科技* 22, 109.

吕志广. (2018). 丹参酮联合缬沙坦对高血压肾病患者氧化应激反应的影响. *临床医学* 38, 108-109. doi: 10.19528/j.issn.1003-3548.2018.08.045

马文涛, 聂芳, 孙桂强, 宋光远. (2017). 丹参酮联合缬沙坦治疗对高血压肾病患者肾损伤、内皮损伤的影响. *海南医学院学报* 23, 1059-1062. doi: 10.13210/j.cnki.jhmu.20170406.064

倪海涛. (2015). 厄贝沙坦联合丹参酮ⅡA磺酸钠治疗高血压合并肾损害患者临床的效果探索. *世界最新医学信息文摘（连续型电子期刊）* 71-71. doi: 10.3969/j.issn.1671-3141.2015.77.045

吐逊姑丽?木沙. (2017). 缬沙坦联合丹参酮注射液治疗64例高血压肾损害的临床疗效观察. *中国医药指南* 15, 3,5.

王琳琳, 卫志锋, 潘星, 陆继芳, 程锦绣, 刘翠兰等. 丹参多酚酸盐联合缬沙坦治疗高血压肾病的临床观察 :河北北方学院附属第一医院,2015.

王琳琳, 卫志锋, 潘星. (2016). 丹参多酚酸盐联合缬沙坦对高血压肾病病人血压及生化指标的影响. *中西医结合心脑血管病杂志* 14, 894-896.

王梦华. (2016). 丹参酮联合缬沙坦治疗高血压肾病的疗效研究. *中国医药指南* 14, 202.

王强, 李超, 周晓花. (2016). 高血压肾病应用丹参酮注射液联合缬沙坦方案的治疗效果分析. *今日健康* 15, 136. doi: 10.3969/j.issn.1671-5160.2016.07.126

杨海燕, 占焕平, 陈华. (2012). 丹参酮与缬沙坦联合治疗高血压肾病疗效观察. *实用医学杂志* 28, 340-341. doi: 10.3969/j.issn.1006-5725.2012.2.075

张建女. (2014). 用丹参酮注射液联合缬沙坦治疗高血压肾病的效果观察. *当代医药论丛* 267-268. doi: 10.3969/j.issn.2095-7629.2014.03.243

朱宏文, 朱明, 高建东. (2011). 丹参酮治疗高血压肾病的临床研究. *内科理论与实践* 06, 204-207.

**REFERENCES (English)**

Chen, K., Zhang, D., Xu, P., Wang, B., Deng, W. (2022). [Medication rule of Chinese medicine in treating early renal damage of hypertension]. *China Medical Herald* 19, 118-122.

Chu, Z., Chen, Z., Jiang, X., Hu, X. (2017). [Effect of salvianolate combined with alprostadil and glutathione on early renal injury of hypertension]. *Cardio-cerebrovascular Disease Prevention and Treatment* 17, 378-380. doi: 10.3969/j.issn.1009_816x.2017.05.17

Hu, S,. (2017). [Effect of tanshinone combined with valsartan on renal function and inflammation in patients with hypertensive nephropathy]. *Research of Integrated Traditional Chinese and Western Medicine* 9, 239-240. doi: 10.3969/j.issn.1674-4616.2017.05.005

Li, Q., Guo, J. (2017). Effect of Tanshinone Injection Combined with Valsartan on Patients with Hypertensive Renal Damage. *Xinjiang Medical Journal* 47, 1304-1305,1308.

Li, S.(2018) [Therapeutic effect of salvianolate, alprostadil and glutathione combined in the treatment of chronic renal insufficiency]. *Chinese Association of Integrated Traditional and Western Medicine Kidney Disease Professional Committee 2018 Annual conference abstract collection*.2018. 1366.

Li, S., Guo, Z., Li, J., Zhao,L. (2016). [Therapeutic effect of salvianolate, alprostadil and glutathione in the treatment of chronic renal failure]. *Chinese Journal of Integrated Traditional and Western Nephrology* 17, 789-790.

Li, T. (2016). [Effect of tanshinone combined with valsartan on hypertensive nephropathy]. *The World Clinical Medicine* 10, 88.

Li, Y. (2014). [Clinical observation of tanshinone injection combined with valsartan in treating 115 cases of hypertensive kidney damage]. *Chinese Journal of Ethnomedicine and Ethnopharmacy* 57-57,58.

Lin, Y. (2020). [Clinical observation of combined salvianolate, alprostadil and glutathione in the treatment of chronic renal failure]. *Guide of China Medicine* 18, 172-173.

Liu,C. (2013). [Protection and nursing of kidney damage of refractory hypertension by combination of traditional Chinese and Western medicine]. *Lishizhen Medicine and Materia Medica Research* 24, 2275-2276. doi: 10.3969/j.issn.1008-0805.2013.09.107

Liu, F. (2018). [Clinical characteristics of hypertensive nephropathy and evaluation of therapeutic effect of tanshinone injection combined with valsartan]. *Cardiovascular Disease Electronic Journal of Integrated Traditional Chinese and Western Medicine* 6, 74. doi: 10.3969/j.issn.2095-6681.2018.13.052

Liu, S., He, Y. (2020). [Study on protection and nursing intervention of Danshen Injection combined with Western medicine on renal damage of refractory hypertension]. *Capital Food Medicine* 27, 130. doi: 10.3969/j.issn.1005-8257.2020.02.105

Liu, Y. (2015). [Treatment of 16 cases with chronic renal failure by salvianolate]. *Chinese Journal of Traditional Medical Science and Technology* 22, 109.

Lv, Z. (2018). [Effect of tanshinone combined with valsartan on oxidative stress in hypertensive nephropathy patients]. *Clinical Medicine* 38, 108-109. doi: 10.19528/j.issn.1003-3548.2018.08.045

Ma, W., Nie, F., Sun, G., Song, G. (2017). Effect of tanshinone combined with valsartan therapy on the renal injury and endothelial injury in patients with hypertensive nephropathy. *Journal of Hainan Medical University* 23, 1059-1062. doi: 10.13210/j.cnki.jhmu.20170406.064

Ni, H. (2015). [Clinical efficacy of irbesartan combined with tanshinone ⅡA sulfonate in the treatment of patients with hypertension complicated with kidney damage]. *World Latest Medicine Information* 71-71. doi: 10.3969/j.issn.1671-3141.2015.77.045

Tu, X. (2017). Clinical Observation of Valsartan combined with Tanshinone Injection in the Treatment of 64 Cases of Hypertensive Renal Damage. *Guide of China Medicine* 15, 3,5.

Wang, L., Wei, Z., Pan, X., Lu, J., Cheng, J., Liu, C. [Clinical observation of salvianolate combined with valsartan in the treatment of hypertensive nephropathy]: The First Affiliated Hospital of Hebei North University,2015.

Wang, L., Wei, Z., Pan, X. (2016). [Effects of salvianolate combined with valsartan on blood pressure and biochemical indices in patients with hypertensive nephropathy]. *Chinese Journal of Integrative Medicine on Cardio-Cerebrovascular Disease* 14, 894-896.

Wang,M. (2016). [Therapeutic effect of tanshinone combined with valsartan on hypertensive nephropathy]. *Guide of China Medicine* 14, 202.

Wang, Q., Li, C., Zhou, X. (2016). [Effect analysis of tanshinone injection combined with valsartan in the treatment of hypertensive nephropathy]. *Journal of Today Health* 15, 136. doi: 10.3969/j.issn.1671-5160.2016.07.126

Yang, H., Zhan, H., Chen, H. (2012). [Effect of tanshinone combined with valsartan on hypertensive nephropathy]. *The Journal of Practical Medicine* 28, 340-341. doi: 10.3969/j.issn.1006-5725.2012.2.075

Zhang, J. (2014). [Effect of tanshinone injection combined with valsartan on hypertensive nephropathy]. *Contemporary Medicine Forum* 267-268. doi: 10.3969/j.issn.2095-7629.2014.03.243

Zhu, H., Zhu, M., Gao, J. (2011). clinical study of hypertensive nephropathy treated with tanshinone. *Journal of Internal Medicine Concepts & Practice* 06, 204-207.
