# Supplementary material for: Salvianolate injection for hypertensive nephropathy patients who were using valsartan: A systematic review and meta-analysis
Source: Front Pharmacol. 2023 Jan 30;14:1119150. doi: 10.3389/fphar.2023.1119150 (PMC9922779; doi:10.3389/fphar.2023.1119150)
Supplement: Supplementary file 1 [file DataSheet1.ZIP › Supplementary_Material.docx]

Supplementary Material

**Supplementary Tables 1.** Search strategies for English databases

**Supplementary Tables 2.** Search strategies for Chinese databases.

**Supplementary Figure 1.** Egger's publication bias plot for blood pressure.

**Supplementary Figure 2.** Egger's publication bias plot for Cystatin C.

**Supplementary Figure 3.** Egger's publication bias plot for Endothelin-1

**Supplementary Figure 4.** Sensitivity Analysis for clinical efficacy

**Supplementary Figure 5.** Sensitivity Analysis for systolic blood pressure

**Supplementary Figure 6.** Sensitivity Analysis for diastolic blood pressure

**Supplementary Figure 7.** Sensitivity Analysis for serum creatinine

**Supplementary Figure 8.** Sensitivity Analysis for urine protein creatinine ratio

**Supplementary Figure 9.** Sensitivity Analysis for cystatin C

**Supplementary Figure 10.** Sensitivity Analysis for endothelin-1

**Supplementary Table 1.** Search strategies for English databases.

| Database name | Search strategies |
| --- | --- |
| [Pubmed](https://pubmed.ncbi.nlm.nih.gov/) | (salvia* AND hypertens* AND (renal OR nephropathy OR kidney)) OR ((salvia*[MeSH Terms]) AND (hypertens*[MeSH Terms]) AND (renal[MeSH Terms] OR nephropathy[MeSH Terms] OR kidney[MeSH Terms])) |
| [Web of science](https://www.webofscience.com/wos/alldb/basic-search) | TS=salvia* AND TS=Hypertens* AND TS=(renal OR nephropathy OR kidney) |
| [Cochrane Library](https://www.cochranelibrary.com/search?cookiesEnabled) | salvia* AND hypertens* AND (renal OR nephropathy OR kidney) in All Text |
| [embase](https://www.embase.com/) | (salvia* AND hypertens* AND ('renal'/exp OR renal OR 'nephropathy'/exp OR nephropathy OR 'kidney'/exp OR kidney) OR ('salvianolate'/exp AND 'hypertensive' AND ('renal'/exp OR 'nephropathy'/exp OR 'kidney'/exp))) |

**Supplementary Table 2.** Search strategies for Chinese databases.

| Database name | Search strategies |
| --- | --- |
| [CNKI](https://www.cnki.net/) | KAT=高血压 AND TKA=丹参多酚 AND (TKA=肾损害 OR TKA=肾病 OR TKA=肾脏损害 OR TKA=肾脏病) |
| [Wanfang](https://www.wanfangdata.com.cn/) | 全部:(丹参多酚) and 全部:(高血压) and 全部:(肾) |
| [VIP](http://cqvip.com/) | U=丹参多酚 AND U=高血压 AND U=肾 |
| [SinoMed](http://www.sinomed.ac.cn/index.jsp) | "丹参多酚"[全部字段:智能] AND "高血压"[全部字段:智能] AND "肾"[全部字段:智能] |

CNKI,China National Knowledge Infrastructure; Wanfang, Wanfang Data knowledge service platform; VIP,China Science and Technology Journal Database; SinoMed,China Biomedical Literature Service System.


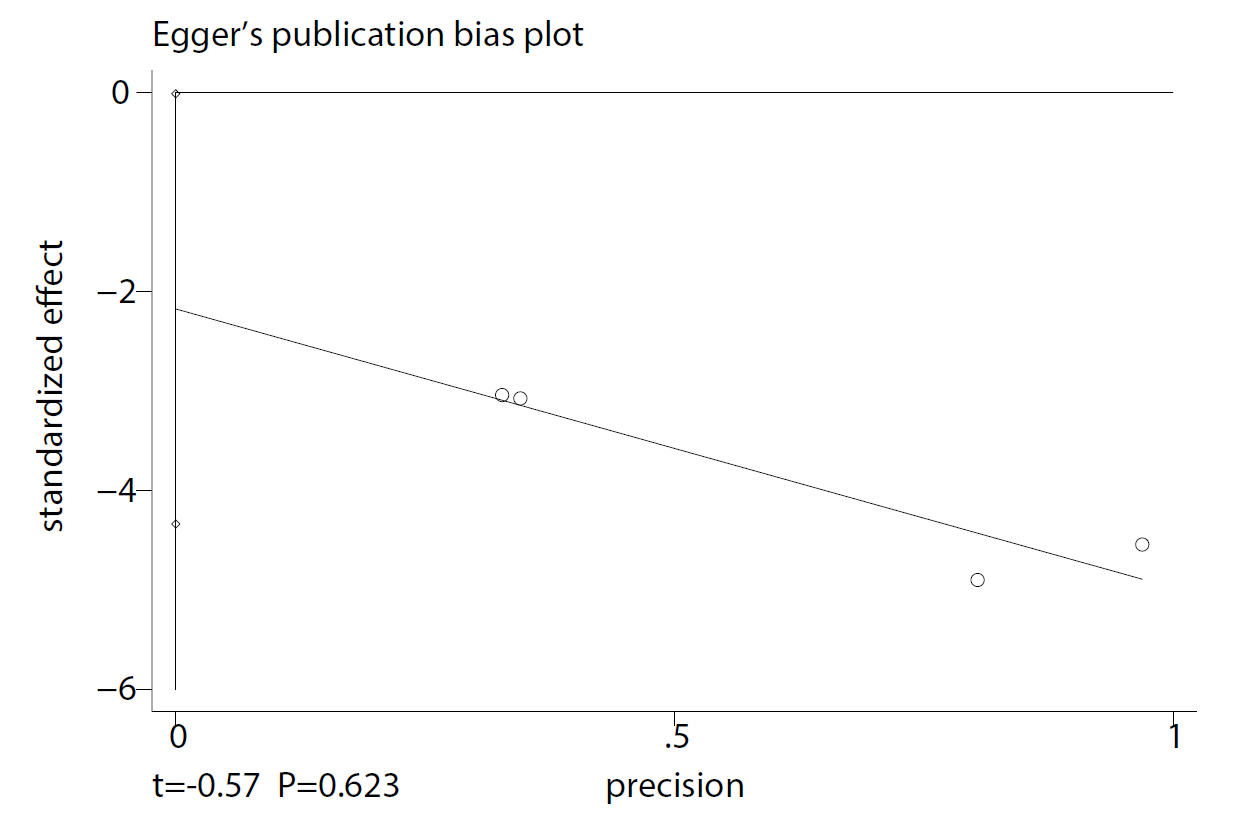


**Supplementary Figure 1.** Egger's publication bias plot for blood pressure.


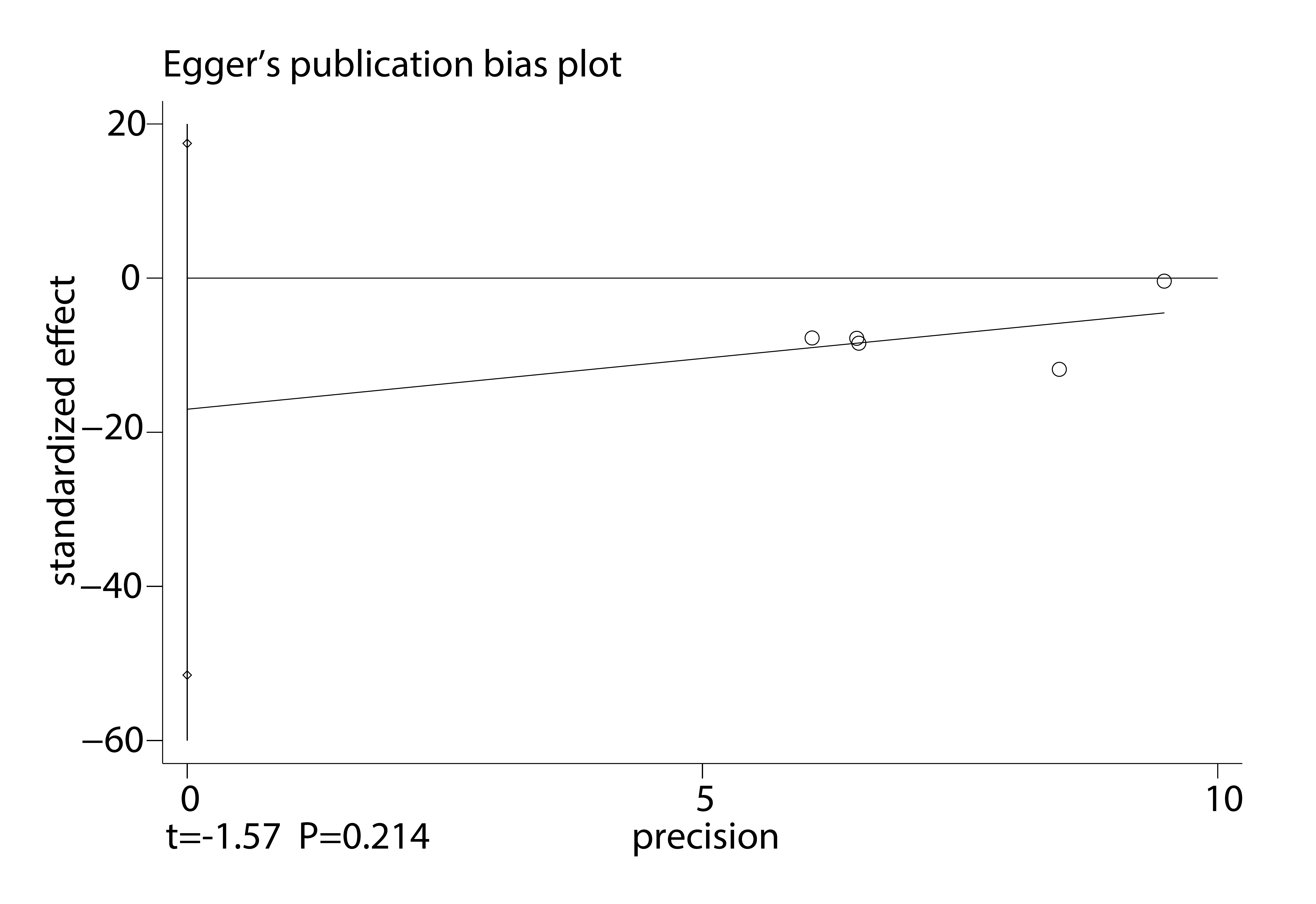


**Supplementary Figure 2.** Egger's publication bias plot for Cystatin C

**Supplementary Figure 3.** Egger's publication bias plot for Endothelin-1

**Supplementary Figure 4.** Sensitivity Analysis for clinical efficacy

**Supplementary Figure 5.** Sensitivity Analysis for systolic blood pressure

**Supplementary Figure 6.** Sensitivity Analysis for diastolic blood pressure

**Supplementary Figure 7.** Sensitivity Analysis for serum creatinine

**Supplementary Figure 8.** Sensitivity Analysis for urine protein creatinine ratio

**Supplementary Figure 9.** Sensitivity Analysis for cystatin C

**Supplementary Figure 10.** Sensitivity Analysis for endothelin-1
